# What insights can ambulance data provide on vulnerable groups?

**DOI:** 10.23889/ijpds.v8i2.2226

**Published:** 2023-09-14

**Authors:** Abigail Brake, Dan Birks, Mark Mon-Williams, Sam Relins

**Affiliations:** 1 University of Leeds, Leeds, United Kingdom; 2 Bradford Institute for Health Research, Bradford, United Kingdom

Linking administrative data from Yorkshire Ambulance Service with primary health care data, this research project aims to answer the question, “What can YAS data tell us about how vulnerable populations interact with the service in Bradford?”

We selected 9 primary callout reasons as recorded in the data that could be vulnerability-related, and explored patterns of these both spatially and temporally, with comparison to all other callout reasons. The data also includes a pseudonymised NHS number which allows linkage with other datasets for which the patient has shared this identifier. In this case, we took their home LSOA to create a rudimentary gravity model visualising flows of people from their home location to their ambulance incident location.

Key findings include that vulnerability-related callouts were more frequent in the evenings and overnight on weekends, and concentrated on specific areas, both in terms of where incidents occur and areas from which callers originate. In terms of the individuals behind the calls, we found that while callers from both subsets were more likely to be female, the average age of callers for vulnerability-related incidents was almost 20 years younger than callers for all other reasons. Additionally, we discovered which callout reasons were most likely to see individuals requiring an ambulance multiple times.

This research provides valuable policy-relevant insights into emergency service demand relating to vulnerable populations in the Bradford region, highlighting the importance of understanding the needs of vulnerable populations to ensure that emergency services are allocated effectively and efficiently.

This study is based [in part] on data from Connected Bradford (REC 18/YH/0200 & 22/EM/0127). The data is provided by the citizens of Bradford and district, and collected by the NHS, DfE and other organisations as part of their care and support. The interpretation and conclusions contained in this study are those of the authors alone. The NHS, DfE and other organisations do not accept responsibility for inferences and conclusions derived from their data by third parties.

